# Bovine Dentin as a Substitute for Human Dentin: Bond Strength Tests on Sound and Eroded Substrate

**DOI:** 10.3390/dj14010066

**Published:** 2026-01-20

**Authors:** Ramona Oltramare, Caroline A. Lutz Guzman, Julia J. Lotz, Thomas Attin, Florian J. Wegehaupt

**Affiliations:** 1 Clinic of Conservative and Preventive Dentistry, Center for Dental Medicine, University of Zurich, 8032 Zurich, Switzerland; thomas.attin@zzm.uzh.ch (T.A.); florian.wegehaupt@zzm.uzh.ch (F.J.W.); 2 Private Practice, 5620 Bremgarten, Switzerland; caroline.lutz@gmx.ch; 3 Private Practice, 81369 Munich, Germany; juliajohannalotz@gmail.com

**Keywords:** eroded dentin, human, bovine, etch-and-rinse, self-etch, micro-tensile bond strength

## Abstract

**Objectives:** Investigating and comparing the micro-tensile bond strength (µTBS) of etch-and-rinse (ER) or self-etch (SE) adhesives on sound (s) and eroded (e) human (H) and bovine (B) dentin. **Methods:** Twenty-four human and bovine teeth were divided into eight groups (*n* = 6) and coronally ground down, exposing their dentin. Two groups of human (HeER + HeSE) and bovine teeth (BeER + BeSE) were subjected to erosive challenges (citric acid (pH 2.7), 10 × 2 min per day for five days, and stored in artificial saliva). Groups HsER + HeER and BsER + BeER were treated with an etch-and-rinse adhesive (OptiBond FL), and groups HsSE + HeSE and BsSE + BeSE were treated with a self-etch adhesive (OptiBond All-in-One), followed by buildups with a composite restorative material. After seven days of storage in tap water, µTBS was determined and failure type analysis was performed. Data were evaluated using two-way ANOVA and Tukey’s post hoc tests at a level of significance of α = 0.05. **Results:** Using etch-and-rinse adhesive, sound human dentin (HsER) showed the significantly highest µTBS (*p* < 0.05) compared to eroded human (HeER) and sound and eroded bovine dentin (BsER + BeER). For sound human and bovine specimens (HsSE + BsSE), there was no significant difference (*p* ≥ 0.05) in µTBS when self-etch adhesive was applied, as well as in the eroded specimens (HeSE + BeSE). **Conclusions:** Within the limitations of this study, it can be concluded that for the etch-and-rinse approach, it is not recommended to substitute human dentin with bovine dentin. When using the specific self-etch adhesive used in the present study, bovine dentin can be used to substitute human dentin, as they showed comparable µTBS.

## 1. Introduction

Over recent decades, the prevalence of caries has declined significantly [1,2,3]. On the other hand, there seems to be an increase in the prevalence of erosive dental hard tissue loss. A recent review [4] reported a prevalence of erosions in 30–50% of deciduous teeth and 20–45% in permanent teeth. Therefore, it is of great importance to treat erosive defects in a long-term and sufficient manner, preferably in a way that is non- or minimally invasive. Modern composite filling materials and adhesive bonding agents enable durable and esthetic restorations. However, bonding to eroded dentin poses a certain challenge [5,6]. Many studies have shown that the bond strength to eroded dentin is considerably lower than to sound dentin [7,8,9,10]. It has been hypothesized that this phenomenon may result from erosive demineralization of dentin upon regular exposure to erosive acids, which leads to the formation of a surface collagen layer. This collagen layer is thought to impede the effective interaction between the dentin substrate and applied adhesive systems [8]. Investigations were carried out to assess which pretreatment steps could be taken to improve the bond strength to the eroded dentin [5,10]. Laboratory studies on eroded tooth structure are ideally carried out on extracted human teeth [11,12,13]. However, improvements in oral health have led to a reduction in tooth loss [14], consequently limiting the availability of extracted teeth for research purposes. In addition, extracted human teeth offer a more difficult comparison since each individual has different dietary and oral hygiene habits (e.g., use of fluoridated oral health products), which cannot be retraced. To counteract these aspects, bovine teeth have been used in several studies [15,16,17,18]. Bovine teeth are usually available in large quantities. When sourced from a single origin, it can be assumed that factors such as nutrition and other environmental influences (e.g., fluoride intake through drinking water) are quite homogeneous. When the erosion and abrasion behavior of human and bovine teeth was investigated, no significant difference was found between the species [19]. A review addressing the question of whether bovine teeth are a suitable substitute in bonding/adhesive investigations of human teeth reported inconsistent results [20]. Some results show equal or even higher values for bovine dentin compared to human dentin [21,22], and some show worse values [23,24,25].

Taking the above-mentioned aspects into consideration, the question of whether bovine dentin is a suitable substitute for human dentin in bonding experiments does not seem to have been conclusively answered. On the one hand, it must be assessed whether the substrate, human and bovine teeth, behaves in the same way when examined in terms of its bond strength to eroded dentin. On the other hand, previous studies have mostly investigated only one material group of adhesives, etch-and-rinse or self-etch adhesives. It is thus difficult, and should even be avoided, to make comparisons between these adhesive types, as it is not recommended to compare the results of different studies of bond strength measurements [26].

Furthermore, to the best of our knowledge, there are no studies that have investigated the use of bovine dentin as a replacement for human dentin in bond strength studies on eroded dentin. Therefore, the aim of the present study was to investigate the suitability of bovine dentin as a substitute for human dentin in a bonding test on sound and eroded dentin when using an etch-and-rinse or a self-etch adhesive. The null hypothesis tested was that there is no difference between human and bovine dentin in terms of adhesion in eroded and sound conditions, whether the etch-and-rinse or self-etch adhesive is used.

## 2. Materials and Methods

### 2.1. Sample Preparation

For this laboratory study, 24 human molars and 24 bovine roots were selected and allocated into eight groups (*n* = 6 per group). The sample size was chosen pragmatically based on feasibility and consistency with a previous study [8] that also performed µTBS tests on eroded dentin. Only human teeth from adult patients (18 years or older) extracted for periodontal reasons were included in the study. Written informed consent was obtained from the patients to use the teeth for research purposes, which were irrevocably anonymized. The bovine dentin samples used were obtained from extracted bovine teeth from a local butchery (SBZ Schlachtbetrieb Zürich AG, Zurich, Switzerland). Under these terms, the project did not fall within the scope of the Human Research Act, and authorization of the project from the Cantonal Ethics Committee was not necessary (BASEC-Nr. Req-2020-00786).

#### 2.1.1. Human Samples

After mechanical cleaning using a scaler, the teeth were stored in 0.2% thymol at 5 °C until use. The teeth were fixated on a scanning electron microscope carrier (Wenka, Kar Wenger SA, Courgenay, Switzerland) using LC Block-Out Resin (Ultradent Products Inc., South Jordan, UT, USA) and embedded in acrylic resin (Paladur, Heraeus Kulzer, Hanau, Germany), covering only the root surface. The crown was trimmed horizontally to the occlusal surface, approximately at the depth of the main groove, with a low-speed precision cutter (IsoMet, Buehler, Lake Bluff, IL, USA). The teeth were then ground with silicon carbide paper (180 grit, Buehler-Met II, Buehler, Lake Bluff, IL, USA) using a polishing machine (Planopol-2, Struers, Ballerup, Denmark) under constant water cooling until no enamel was left in the central part. Using a stereomicroscope (Stemi 1000, Carl Zeiss, Feldbach, Switzerland), the center of the samples was checked for remnants of enamel or pulp exposure. Samples obtained from human teeth were allocated to four groups (HsER, HeER, HsSE, and HeSE). The group codes were defined as follows: the first capital letter indicates the substrate (H = human, B = bovine), the subsequent lowercase letter specifies the dentin condition (s = sound, e = eroded), and the final letters refer to the adhesive system applied (ER = etch-and-rinse adhesive, SE = self-etch adhesive).

#### 2.1.2. Bovine Samples

**Figure 1:**
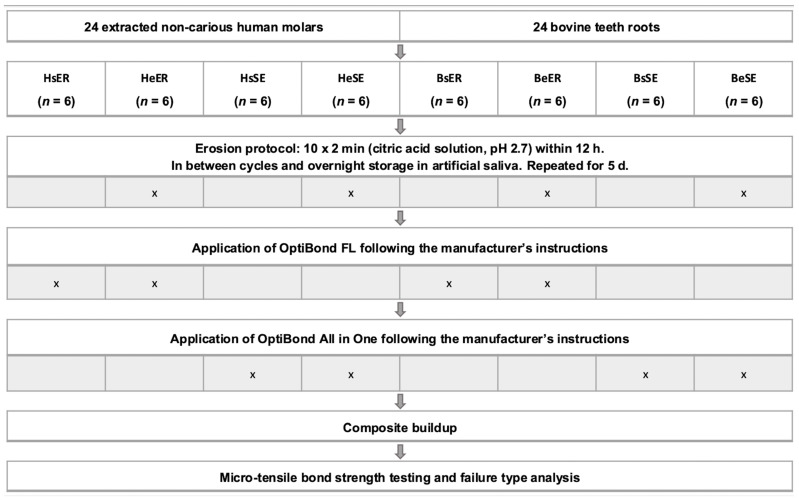
Sample allocation and experimental protocol.

Bovine teeth were used immediately after extraction. The teeth were cleaned, and the crown was separated from the root at the cementoenamel junction using the low-speed precision cutter (IsoMet, Buehler, Lake Bluff, IL, USA). After removing the pulp, the roots were fixated vertically lying on the SEM carriers (Wenka, Kar Wenger SA, Courgenay, Switzerland) using LC Block-Out Resin (Ultradent Products Inc., South Jordan, UT, USA) and embedded in acrylic resin (Paladur, Heraeus Kulzer, Hanau, Germany). It was ensured that the most even surface of the root was facing upwards. The embedded samples were ground with 180-grit silicon carbide paper under continuous water cooling as described above until the curved root surface was planar. Samples gained from bovine teeth were also allocated to four groups (BsER, BeER, BsSE, and BeSE). Figure 1 shows the allocation of the groups and the experimental protocol.

### 2.2. Erosive Citric Acid Cycling

Two groups, each with human and bovine teeth, were subjected to erosive challenges. The remaining groups represented the respective control groups (sound dentin) and were stored in tap water during the erosion challenges of the test groups. Following the protocol of Deari et al. [8], the samples of groups HeER, HeSE, BeER, and BeSE were completely submerged in citric acid solution (pH 2.7; Merck, VWR International, Zurich, Switzerland) and eroded for 2 min on a shaking table (VXR basic Vibrax, IKA, Staufen, Germany) with constant agitation.

After the erosive challenge, the teeth were rinsed with tap water for 10 s and stored in artificial saliva (pH 6.4) under agitation for remineralization at room temperature for 45 min. After two cycles, the artificial saliva was changed. This procedure was repeated ten times daily for five days. Overnight, the teeth were also stored in artificial saliva. In total, each sample was eroded for 100 min. Both the citric acid and the artificial saliva were prepared in-house. The composition of the artificial saliva is according to Klimek et al. [27] but without the addition of ascorbic acid and D + glucose to ensure a longer preservability.

### 2.3. Restoration

A transparent ring matrix (Lucifix Matrices, Kerr, Orange, CA, USA) was placed around the teeth. Groups HsER, HeER, BsER, and BeER were treated with OptiBond FL (Kerr, Orange, CA, USA) according to the manufacturer’s instructions. The dentin was etched with 37.5% phosphoric acid (Gel Etchant, Kerr, Orange, CA, USA) for 15 s and thoroughly rinsed with water for 15 s. After the dentin surface was blown with oil-free air for 3 s, the primer (OptiBond FL, Kerr, Orange, CA, USA; LOT: 7290199) was rubbed in for 15 s with a microbrush and lightly blown for 5 s. The adhesive (OptiBond FL, Kerr, Orange, CA, USA; LOT: 7290194) was also rubbed in for 15 s, blown for 3 s, and light-cured using an LED curing unit (Bluephase G2, Ivoclar Vivadent, Schaan, Liechtenstein; radiant exitance: 1200 mW/cm^2^) for 20 s, as close and perpendicular as possible. A Bluephase meter (Ivoclar Vivadent AG, Schaan, Liechtenstein) was used to measure the output irradiance, which was checked at regular intervals.

Groups HsSE, HeSE, BsSE, and BeSE were treated with OptiBond All-in-One (Kerr, Orange, CA, USA; LOT: 1923187) according to the manufacturer’s instructions. The adhesive was applied to the dentin surface twice in succession for 20 s each and blown for 5 s after the second application. The adhesive was then light-cured for 10 s with the aforementioned light-curing device.

Both adhesive systems used in this study, including their compositional details as specified by the manufacturer, are given in Table 1.

After treatment with the respective adhesives, composite buildups (Ceram.x Spectra ST (HV), Dentsply Sirona, Konstanz, Germany; shade A3; LOT: 1810000999) of three increments with a thickness of 2 mm each were performed. Each increment was polymerized for 20 s. Until further use, the samples were stored in tap water at 37 °C for one week. This study utilized tap water from Zurich with a pH value of 7.91, a conductivity at 20 °C of 302 µS/cm, a water hardness of 14–19 °fH (7–10 °dH), and a calcium content of approximately 51.8 mg/L and a magnesium content of 7.6 mg/L [28]. The number of aerobic, mesophilic bacteria (CFU) in the distribution network was well below the legal maximum of 100 CFU/mL [28].

### 2.4. Micro-Tensile Bond Strength Test

The micro-tensile bond strength (µTBS) test was performed according to the protocol of Armstrong et al. [29]. The samples were cut longitudinally in two directions using a water-cooled diamond saw (Accutom-50, Struers, Ballerup, Denmark) with a diamond wheel (M0D10, Struers, Ballerup, Denmark; diameter: 102 mm, thickness: 0.3 mm). Subsequently, the samples were cut parallel to the surface of the tooth with a low-speed precision cutter (IsoMet, Buehler, Lake Bluff, IL, USA) to obtain nine rectangular sticks from the central portion of each tooth. Using a stereomicroscope (Stemi 1000, Carl Zeiss, Feldbach, Switzerland), it was ensured that there were no enamel remnants on the sticks. Each stick was measured with a digital micrometer (406-250-30, Mitutoyo AG, Urdorf, Switzerland), and the bonding surface area was calculated, which had a mean of 0.83 ± 0.04 mm^2^. The sticks were glued (cyanoacrylate glue; Renfert GmbH, Hilzingen, Germany) into previously sandblasted (50 µm aluminum oxide) µTBS jigs (Wenka, Karl Wenger SA, Courgenay, Switzerland). Thus, the prepared sticks were placed in the universal testing machine (Zwick Roell Z010, Ulm, Germany) and loaded under tension until failure with a crosshead speed of 1 mm/min, using a load cell of 500 N. To calculate the µTBS (MPa), the load at failure (N) was divided by the respective bonding area (mm^2^). Lastly, the tested sticks were examined under a stereomicroscope (Stemi 1000, Carl Zeiss AG, Oberkochen, Germany) at 10× magnification to determine the type of failure.

### 2.5. Scanning Electron Microscope Imaging

For each experimental group, one additional specimen was prepared to obtain representative scanning electron microscope (SEM) images of the failure modes. These specimens were not included in the µTBS testing and therefore serve only an illustrative purpose. After testing, the fractured sticks were thoroughly dried and mounted on SEM carriers (Wenka, Kar Wenger SA, Courgenay, Switzerland) using carbon pads. The specimens were then coated with Leit-C at the margins and sputter-coated with a 10 nm gold layer. SEM imaging was performed using a field-emission scanning electron microscope (GeminiSEM 450, Carl Zeiss AG, Oberkochen, Germany) equipped with an Everhart–Thornley secondary electron detector. Imaging was conducted at an acceleration voltage of 10 kV in high-vacuum mode. The working distance ranged between 5.5 and 7 mm. The beam current was set to 200 pA, while the spot size was controlled automatically by the system. Images were acquired at a nominal magnification of 500×.

### 2.6. Statistical Analysis

µTBS values of specimens that failed prior to testing (pre-test failure: PTF) were set to 0 MPa [29]. Data were analyzed using the statistical software R 4.0.0. [30] and the tidyverse package [31]. For each tooth, the µTBS values of the nine sticks were averaged to calculate the mean value. This resulted in six values per group. To investigate the influence of substrate (human vs. bovine) and condition (sound vs. eroded), the data set was split according to the respective adhesive (etch-and-rinse or self-etch). Therefore, two separate 2-way ANOVAs (Type III) and Tukey’s post hoc tests were performed. Model assumptions were evaluated using residual diagnostics (Q–Q plots, Residuals-vs.-Fitted plots, and Scale–Location plots). The residuals showed no substantial deviations from normality or homoscedasticity. The level of significance was set at α = 0.05.

## 3. Results

### 3.1. µTBS for Etch-and-Rinse Adhesive

**Figure 2:**
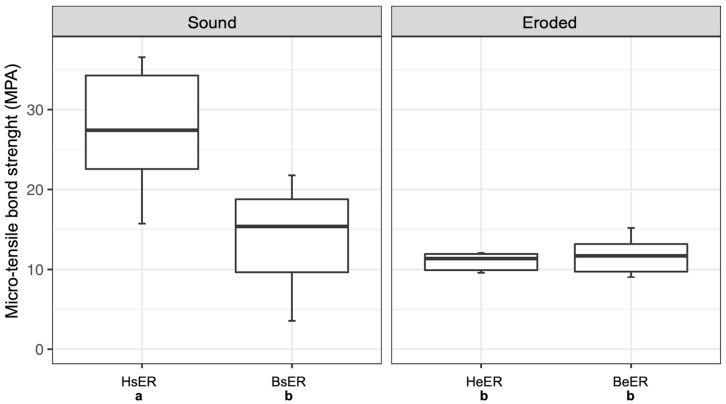
Micro-tensile bond strength (µTBS, in MPa) of sound and eroded human and bovine dentin using the etch-and-rinse adhesive. Boxplots show the medians (black lines) with 25 and 75% quartiles (boxes). The whiskers represent 1.5  ×  interquartile range (IQR), or minima and maxima of the distribution if below 1.5  ×  IQR. µTBS values that were not significantly different (*p* ≥ 0.05) are marked with the same letter. Different letters indicate significant differences (*p* < 0.05) between µTBS values.

Figure 2 presents the µTBS of sound and eroded human and bovine dentin when using the etch-and-rinse adhesive. While sound human dentin (HsER; mean ± SD: 25.16 ± 11.19 MPa) showed significantly higher µTBS values, no significant differences were observed among eroded human dentin (HeER; 9.04 ± 3.17) and bovine dentin, whether sound (BsER; 11.78 ± 7.56) or eroded (BeER; 11.32 ± 2.81). Table 2 shows the corresponding ANOVA-output for the etch-and-rinse adhesive.

**Figure 3:**
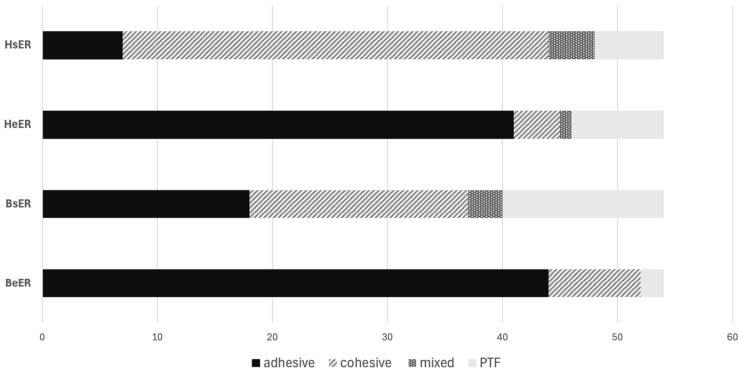
Failure type analysis for groups pretreated with the etch-and-rinse adhesive.

Figure 3 shows the failure mode distribution for sound and eroded human and bovine dentin when using the etch-and-rinse adhesive. In both human and bovine substrates, the incidence of adhesively failed sticks increased in eroded dentin (HeER and BeER) compared to sound dentin (HsER and BsER).

### 3.2. µTBS for Self-Etch Adhesive

**Figure 4:**
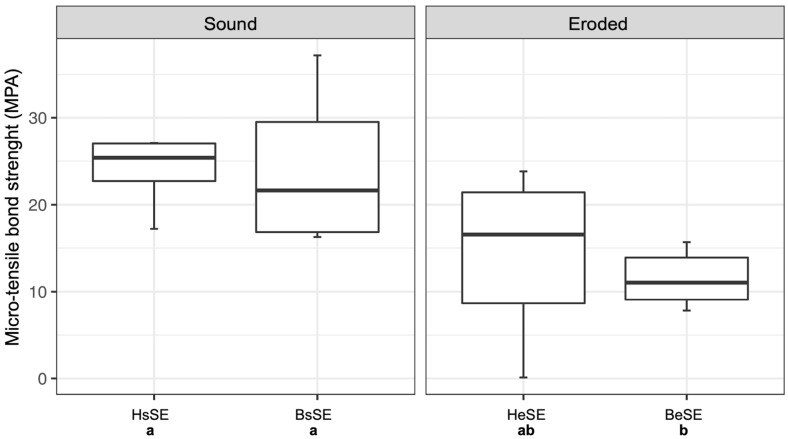
Micro-tensile bond strength (µTBS, in MPa) of sound and eroded human and bovine dentin using the self-etch adhesive. Boxplots show the medians (black lines) with 25 and 75% quartiles (boxes). The whiskers represent 1.5  ×  interquartile range (IQR), or minima and maxima of the distribution if below 1.5  ×  IQR. µTBS values that are not significantly different (*p* ≥ 0.05) are marked with the same letter. Different letters indicate significant differences (*p* < 0.05) between µTBS values.

The µTBS values of sound and eroded human and bovine dentin when using the self-etch adhesive are shown in Figure 4. Sound human (HsSE; 25.36 ± 5.89) and sound bovine dentin (BsSE; 22.91 ± 9.31) showed no statistically significant difference. The two eroded groups also showed no significant difference. Eroded bovine dentin (BeSE; 10.67 ± 4.07) showed significantly lower µTBS values than sound dentin. Human eroded dentin (HeSE; 13.56 ± 9.38) had lower µTBS values than sound dentin, but showed no significant difference to either sound dentin substrates or eroded bovine dentin. The corresponding ANOVA-output is shown in Table 3 for the self-etch adhesive.

**Figure 5:**
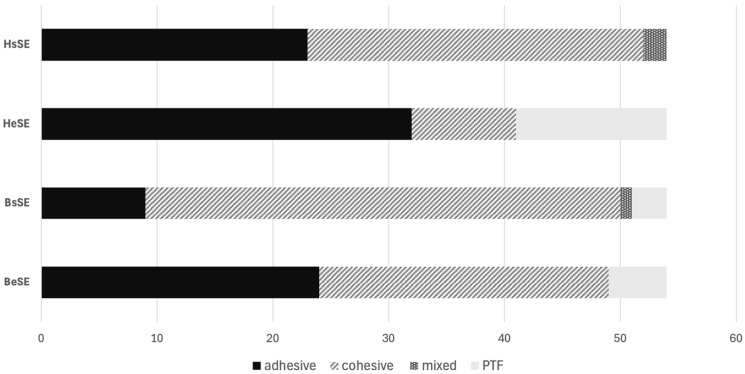
Failure type analysis for groups pretreated with the self-etch adhesive.

Figure 5 shows the failure mode distribution for sound and eroded human and bovine dentin when using the self-etch adhesive. The incidence of adhesively failed sticks also increased from sound (HsSE and BsSE, respectively) to eroded dentin (HeSE and BeSE, respectively) in both substrates when self-etch adhesives were used. However, adhesive and cohesive failures were equally present in eroded bovine dentin (BeSE).

### 3.3. SEM Images

**Figure 6:**
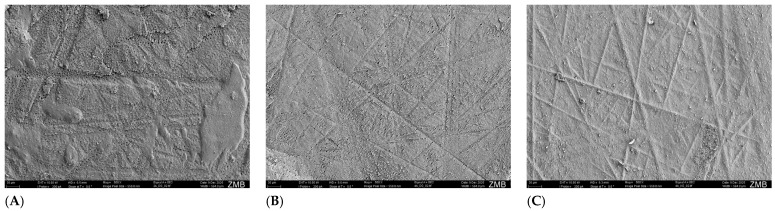
Adhesive failure types. (**A**): human dentin; (**B**): bovine dentin; (**C**): composite.

**Figure 7:**
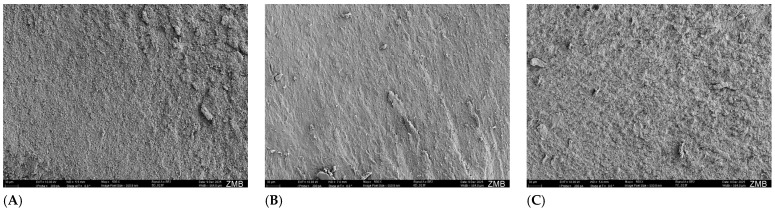
Cohesive failure types. (**A**): human dentin; (**B**): bovine dentin; (**C**): composite.

**Figure 8:**
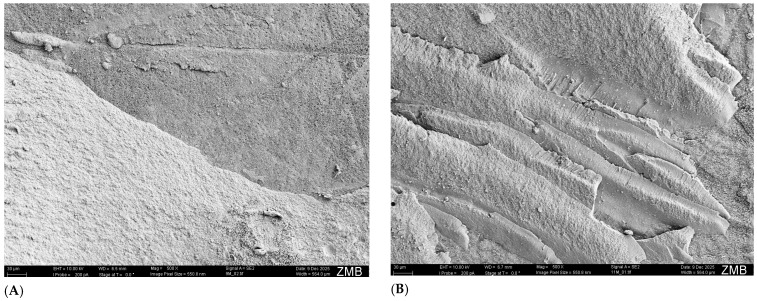
Mixed failure types. (**A**): human specimen, dentin top right of the image, composite bottom left of the image; (**B**): bovine specimen, dentin right in the image, composite left in the image.

Figure 6, Figure 7 and Figure 8 show representative SEM images of the analyzed failure modes of human and bovine substrates.

The dentin side of the adhesive fractures (Figure 6A,B) shows clear grinding marks from the sample preparation. The dentinal tubules are clearly visible and appear larger and denser in human dentin (Figure 6A) than in bovine dentin (Figure 6B). The composite side of the adhesive fractures (Figure 6C) shows the negative imprint of the grinding marks. The resin tags that detached from the dentinal tubules, together with the adhesive layer during the µTBS examination, are clearly visible.

The view of the cohesive fractures in the dentin once again shows the different number of dentinal tubules in human (Figure 7A) and bovine dentin (Figure 7B).

## 4. Discussion

In this study, human and bovine dentin were compared in their bond strength. In detail, it was investigated whether these two substrates behave the same on eroded and sound conditions when treated with an etch-and-rinse (OptiBond FL) or self-etch (OptiBond All-in-One) adhesive. The stated null hypothesis had to be rejected, as sound human and sound bovine dentin differed significantly when the etch-and-rinse adhesive was applied. However, no significant differences were found between human and bovine dentin when the self-etch adhesive was used.

Dentin bond strength was measured using the µTBS methodology. This is a standardized methodology to determine the adhesion to tooth structure, but at the same time, it is much criticized. Not only is this methodology very application-sensitive, but it should be performed accurately by the user. Nevertheless, high standard deviations can occur, as also occurred partly in this study. Furthermore, µTBS does not represent a one-to-one clinically transferable situation. However, even if µTBS cannot be directly equated with the clinical situation, useful conclusions can still be drawn about the clinical performance of an investigated material if performed and interpreted correctly [26].

The adhesives used in the present study differed in their mode of application (etch-and-rinse and self-etch). OptiBond FL was considered the gold standard of etch-and-rinse adhesives for a long time [32,33]. To remain in the same product line, OptiBond All-in-One was chosen for the self-etch adhesive, which originates from the same manufacturer.

In this study, only the influences of extrinsic erosion were investigated, not of intrinsic erosion. The simulation of intrinsic erosions would require enzymes such as trypsin or pepsin found in gastric juice, which were not used in the present investigation. Such enzymes possess proteolytic properties that could dissolve dentinal collagen over time [34]. In the present study, a pure acid challenge was performed in which the collagen layer was not removed. Thus, the worst-case scenario of erosion was investigated with regard to the bond strength test, because it is known that a pronounced collagen network reduces the bonding ability of the adhesive to the underlying undemineralized dentin [35,36].

The results of the investigations on human teeth confirmed the reduction in the bond strength on eroded dentin compared to sound dentin for both adhesive systems, which has already been demonstrated in numerous studies [9,37,38]. These results were also reflected in the failure modes. Cohesive fractures were found mainly on sound dentin, whereas with decreasing µTBS on eroded dentin, the number of adhesive failures and PTFs increased, indicating a weakened adhesive interface. Continual erosive challenges lead to a thick demineralized dentin zone [35]. The exposed collagen fibrils prevent thorough infiltration of the adhesive and thus lead to an impaired hybrid layer, which consequently, reduces the µTBS of the composite to dentin [16,35,36,39].

When considering the results of the present study, there was a significant reduction in the bond strength of eroded dentin (HeER) compared to sound dentin (HsER) when using the etch-and-rinse adhesive on human teeth. A similar tendency of decrease in µTBS was observed when using the self-etch adhesive on human dentin (HsSE and HeSE), although the results were not statistically significant. Even though the etch-and-rinse adhesive showed higher µTBS on sound dentin, the self-etch adhesive appeared to be less affected in its bond strength when used on eroded dentin. These findings have been confirmed in previous studies [8,10,36,40,41]. It can be assumed that additional etching with phosphoric acid and the associated rinsing of the dentin could dissolve any remaining hydroxyapatite, resulting in the complete collapse of the collagen fibrils [10,40,41]. When applying self-etch adhesives, no prior etching is required. The hydroxyapatite crystals around the dentinal tubules can be incorporated into the hybrid layer, which presumably stabilizes the collagen network [10,40].

Sound bovine dentin (BsER) exhibited significantly poorer µTBS than sound human dentin (HsER) when the etch-and-rinse adhesive was applied. Failure mode analysis also showed different distribution patterns. In bovine dentin, adhesive fractures and PTFs were more prevalent than in human dentin. It seems that bovine dentin cannot achieve the same high bond strength when treated with etch-and-rinse adhesives as human dentin. However, there was no difference in bond strength to eroded bovine dentin (BeER). The known decrease in bond strength in human substrate from sound to eroded dentin when using etch-and-rinse adhesives was not reproduced in the bovine substrate. In eroded dentin, not only does the µTBS show no significant differences compared to human dentin, but the fracture analysis also showed a similar distribution pattern with a majority of adhesive fractures.

The application of the self-etch adhesive, on the other hand, showed a similar behavior pattern for both substrates. This applied to both the µTBS and the failure mode analysis, although bovine dentin had a higher proportion of cohesive fractures than human dentin. In sound dentin, there was no significant difference in µTBS between human (HsSE) and bovine teeth (BsSE). Also, eroded dentin (HeSE and BeSE) showed no significant difference between the two substrates.

No other study has yet compared human and bovine teeth in terms of their µTBS on sound and eroded dentin. Studies on bovine teeth investigating etch-and-rinse and 2-step self-etch adhesives show similar trends of µTBS on eroded dentin as in the present study, although these findings were not statistically significant [17,18]. Bovine dentin appears to be somewhat histologically different from human dentin [42,43,44]. Not only does bovine dentin have fewer dentinal tubules compared to human dentin, but the radii were also smaller [45]. This tendency was also evident in the SEM images obtained in the present study (Figure 7A,B). Because of these differences, bovine dentin may be more susceptible to phosphoric acid etching on sound dentin. Possibly, the hydroxyapatite crystals dissolve more easily from sound dentin, resulting in a more pronounced collagen network, which prevents the proper infiltration of the adhesive. Consequently, a less stable hybrid layer is formed, leading to reduced µTBS compared to human dentin. It is hypothesized that this effect is further enhanced by the higher proportion of intertubular dentin in bovine dentin. When etch-and-rinse adhesives are used, a larger area of intertubular dentin is exposed, leading to greater demineralization and excessive exposure of collagen. In human dentin, larger and denser dentinal tubules mean that there is less intertubular dentin; etching and rinsing expose sufficient collagen for an effective hybrid layer, while the numerous tubules allow proper formation of resin tags, resulting in strong adhesion. In contrast, bovine dentin has fewer and smaller tubules, possibly resulting in fewer and shorter resin tags, which might lead to lower dentin bond strength.

Supporting this theory, etch-and-rinse adhesives have shown longer resin tag lengths and more pronounced hybrid layer thicknesses on human sound dentin compared to self-etch adhesives [6].

Self-etching adhesives, which do not require prior etching with phosphoric acid, still perform well on human dentin due to sufficient resin tag formation. On bovine dentin, self-etch adhesives may perform better than etch-and-rinse adhesives because the milder acidic components partially demineralize the intertubular dentin, potentially resulting in a more stable hybrid layer and improved bond strength. In this study, the self-etch adhesive OptiBond All-in-One, with a pH of approximately 2.5, can be classified as a mild adhesive, which may help to explain the observed bonding behavior [46].

Not only the application mode (etch-and-rinse vs. self-etch), but also the viscosity of the two different adhesive systems may help explain the divergent results observed on human and bovine dentin. Etch-and-rinse adhesives, following phosphoric acid etching, may produce a more impermeable collagen layer and, due to their higher viscosity resulting from increased filler content, can also show reduced penetration into the smaller dentinal tubules in bovine dentin. This is particularly relevant for OptiBond FL, as the adhesive contains a relatively high filler fraction of 48 wt% [47]. In contrast, self-etch adhesives may benefit not only from their milder conditioning effect but also from their lower viscosity, related to higher solvent content and the presence of more hydrophilic monomers, which appear to facilitate penetration through the collagen layer into smaller tubules [47,48]. These characteristics may contribute to improved bond strength in bovine dentin compared with etch-and-rinse adhesives.

The distribution of PTFs supports the hypotheses proposed above, as PTFs occurred particularly frequently in groups BsER and HeSE. In the BsER group, phosphoric acid etching likely resulted in a thinner or partially demineralized hybrid layer, leading to excessive exposure of collagen fibers and an increased occurrence of PTFs. In the HeSE group, the absence of phosphoric acid etching may have limited resin infiltration into the eroded dentin, similarly contributing to a higher frequency of PTFs.

In the present study, only one adhesive of the respective adhesive system was investigated at a time. Both adhesives contained GPDM as the functional monomer. Further investigations are necessary to clarify whether the behavior patterns found here generally apply to both adhesive systems. If the present results are confirmed in further studies using other adhesive formulations, for example, containing 10-MDP or 4-META, this could provide additional insight into whether the observed substrate-dependent differences are consistent across adhesives with different chemical bonding mechanisms. Future studies should also include morphological and chemical analyses (e.g., SEM images of longitudinal sections or FTIR) to better understand the substrate–adhesive interactions at the microstructural and molecular levels. It should also be acknowledged that the present study did not include an independent validation of the erosively altered substrate (e.g., surface microhardness, profilometry), which limits the findings to a descriptive assessment of bond strength. Ultimately, this may indicate that the histological differences between human and bovine dentin are too substantial to allow general substitution of one for the other in bond strength tests.

Further limiting the present study is the use of different anatomical regions for human and bovine dentin samples (coronal vs. radicular). These regions differ in terms of tubule density, diameter, and permeability, which may influence adhesive performance regardless of substance type [42,49]. However, the selection is consistent with standard practice in µTBS studies, as coronal human and radicular bovine dentin provide sufficiently large and flat bonding surfaces that are suitable for sample preparation.

Another limitation relates to the choice of the storage media during the erosive and post-restorative phases. The storage medium used during the erosive challenge was artificial saliva, as this allowed the intended remineralization effects between the citric-acid cycles. In contrast, the subsequent seven-day storage after restoration was performed in tap water. Since this period served only as a short-term post-restorative storage without the aim of inducing additional chemical effects, tap water was considered sufficient. Nevertheless, future studies should ideally use artificial saliva for this post-restorative storage phase as well, to standardize the storage conditions throughout the entire experimental procedure.

Moreover, the specimens were stored in tap water for only one week after buildup before µTBS tests were performed. Therefore, this study focused on the short-term storage. Follow-up studies could be conducted based on the results of this study to investigate the effects of long-term storage of µTBS on eroded human and bovine dentin.

## 5. Conclusions

Within the scope and limitations of this study, the following may be concluded:

Etch-and-rinse adhesive

- The µTBS differs significantly in sound human and bovine dentin.
- It does not reflect the behavioral pattern of human substrate when used on bovine dentin.
- Thus, values recorded for the bovine substrate should not be compared equally with human substrate.

Self-etch adhesive

- Within the specific self-etch protocol and materials used here, bovine and human dentin showed comparable µTBS in sound and eroded dentin.
- Accordingly, the human substrate can be replaced by a bovine substrate when using the self-etch adhesive used in the present study.

However, this does not establish general substitutability across self-etch systems or test conditions. Further studies need to be conducted to confirm these results with other adhesives and after long-term storage.

## Figures and Tables

**Table 1:** Composition of the adhesive systems used in this study, as provided by the manufacturer.

Product	Manufacturer	Type	Composition	pH	LOT
OptiBond FL	Kerr Orange, CA, USA	3-step etch-and-rinse	Primer: HEMA ^1^, GPDM ^2^, CQ ^3^, BHT ^4^, water, ethanol	2.9–3.0	7290199
			Adhesive: bis-GMA ^5^, HEMA, GPMA ^6^, CQ, ODMAB ^7^, silica fillers, coupling factor A174	neutral	7290194
OptiBond All-in-One	Kerr Orange, CA, USA	1-step self-etch	GPDM, HEMA, GDMA, bis-GMA, water, ethanol, acetone, CQ, coupling factor A174	~2.5	1923187

^1^ HEMA: 2-hydroxylethyl methacrylate; ^2^ GPDM: glycerophosphate dimethacrylate; ^3^ CQ: camphor quinones; ^4^ BHT: butylhydroxytoluene; ^5^ Bis-GMA: bisphenol-A-glycidyl-dimethacrylate; ^6^ GPMA: Glycidyl Methacrylate; and ^7^ ODMAB: octadecyl dimethyl ammonium bromide.

**Table 2:** Two-way Type-III Anova for the etch-and-rinse adhesive.

Effect	df1	df2	F	*p*	Partial η^2^	95% CI Lower Limit	95% CI Higher Limit
Substrate (human vs. bovine)	1	20	3.70	0.069	0.156	0.000	1
Condition (sound vs. eroded)	1	20	8.23	0.009	0.292	0.050	1
Substrate × condition	1	20	7.35	0.013	0.269	0.038	1

**Table 3:** Two-way Type-III ANOVA for the self-etch adhesive.

Effect	df1	df2	F	*p*	Partial η^2^	95% CI Lower Limit	95% CI Lower Limit
Substrate (human vs. bovine)	1	20	0.756	0.395	0.036	0.000	1
Condition (sound vs. eroded)	1	20	15.341	0.001	0.434	0.159	1
Substrate × condition	1	20	0.005	0.945	0.000	0.000	1

## Data Availability

The original contributions presented in this study are included in the article. Further inquiries can be directed to the corresponding author.
